# Clinician Perspectives on Ambient AI Scribes in the Intensive Care Unit: Qualitative Interview Study

**DOI:** 10.2196/81445

**Published:** 2026-07-02

**Authors:** Laleh Jalilian, Navid Manafi, Matthew Scott Vandiver, Paul Lukac, Achuta Kadambi

**Affiliations:** 1 Ronald Reagan UCLA Medical Center, 757 Westwood Plaza, Suite 3325, Los Angeles, CA, 90049, United States, 1 (310) 267-6629; 2 Department is Electrical and Computer Engineering, University of California, Los Angeles, Los Angeles, CA, United States

**Keywords:** ambient artificial intelligence, ambient AI scribes, AI-generated documentation, intensive care unit, ICU documentation, human-computer interaction, quality improvement, transparency and trust in AI

## Abstract

**Background:**

In intensive care unit (ICU) settings, structured team-based communication, such as multidisciplinary rounds, handoffs, and goals-of-care discussions, is foundational to high-quality care. However, accurately documenting these complex discussions in the medical record remains a challenge due to time pressures, documentation burdens, and competing clinical demands. Ambient artificial intelligence (AI) scribes, which passively transcribe and summarize spoken interactions, offer a potential solution to assist ICU clinicians with documentation. Yet, little is known about how ICU clinicians perceive the integration of these tools into their high-stakes, collaborative workflows.

**Objective:**

This study explores clinicians’ perceptions of integrating ambient AI scribes into structured team-based ICU discussions, including multidisciplinary rounds, handoffs and transitions of care, and goals-of-care discussions, with the broader goal of informing the implementation of these scribes into real-world ICU clinical workflows.

**Methods:**

Interviews and focus groups were conducted with ICU clinicians, including nurses, attendings, trainees (residents/fellows), respiratory therapists, and advanced practice practitioners, who routinely participate in structured ICU discussions. Transcripts were analyzed using grounded theory to identify documentation needs, barriers to documentation, and considerations for the implementation of ambient AI scribes in the ICU setting.

**Results:**

A total of 52 individuals, including 18 ICU attendings, 5 advanced practice practitioners, 10 ICU trainees, 9 ICU nurses, and 10 ICU respiratory therapists, participated. Clinicians emphasized the importance of accurate documentation, but noted persistent barriers such as time constraints, documentation burden, and competing teaching and patient care responsibilities. Clinicians expressed enthusiasm about ambient AI scribes’ potential to reduce documentation burden and improve quality, but requested personalization of outputs, robust consent protocols, and transparency around data use. Participants viewed ambient AI scribes as a promising tool to enhance both documentation fidelity and communication quality in ICU settings. Successful implementation may be contingent upon transparent data governance, specialty-specific customization, and sustained efforts to build clinician trust.

**Conclusions:**

ICU clinicians were optimistic about the potential of ambient AI scribes to ease documentation burden and improve the capture of critical clinical discussions, but expressed concerns over transparency regarding data use. Successful implementation may depend on clinician training, customization of output, and transparent institutional policies on data use and consent.

## Introduction

In the intensive care unit (ICU), structured team discussions, such as multidisciplinary daily rounds, handoffs and transitions of care, and goals-of-care discussions, are essential for coordinating care and conveying critical information. However, despite their importance, a major limitation of these communication workflows is that what is verbally discussed may not be comprehensively or even formally documented in the electronic health record (EHR) [1,2]. These discussions contain clinically rich information that should be documented with high fidelity, but documenting these discussions is often suboptimal due to a confluence of factors, including the time-intensive and cognitively demanding nature of summarizing complex discussions and balancing documentation with direct patient care and trainee teaching. For example, multiple studies have highlighted systemic challenges in documenting goals-of-care discussions with sufficient clarity and depth, raising concerns about medico-legal clarity [3-5]. Similar concerns extend to ICU progress notes, where documentation quality may be variable [6-9], and to handoffs and transitions of care, which, despite their clinical significance, are not routinely documented at most institutions. This documentation gap threatens information continuity and patient safety.

Recent advances in large language models and ambient computing have led to the development of ambient artificial intelligence (AI) scribes, which are voice-enabled AI systems that passively listen to clinical conversations and generate draft notes. To date, fully autonomous, EHR-integrated scribes have been used primarily in the ambulatory outpatient setting, with improvements in physician task load, burnout, and usability [10-12]. While prior work has explored the impact of this technology on patient-provider interactions primarily in ambulatory settings, an unexplored opportunity lies in utilizing ambient AI scribes to enhance structured provider-to-provider communication and documentation in ICU settings. With the prevalence of large, multidisciplinary discussions, multiple speakers, and medically complex terminology, the ICU presents unique challenges for deployment and serves as a meaningful testbed for this technology in inpatient workflows. However, there is limited empirical evidence examining ICU teams’ perceived need for integrating ambient AI scribes into clinical workflows. Further, the passive capture of team-based conversations introduces complex ethical and operational considerations, particularly given the medico-legal implications of these discussions and the potential for ambient AI scribe systems to hallucinate, generate errors in noisy, multispeaker ICU environments, and exhibit bias. While common mitigation strategies include human-in-the-loop review, the use of structured templates, and chart retrieval, these approaches have not been well studied in inpatient settings, where communication is often provider-to-provider and documentation structures differ substantially from ambulatory care.

In this paper, we present findings from a qualitative study involving semistructured interviews with ICU clinicians across multiple roles. The contributions of our work include (1) insights into ICU clinicians’ expectations, concerns, and aspirations regarding the use of ambient AI scribes for documenting common structured, team-based discussions and (2) a set of implementation implications and sociotechnical considerations for safely and meaningfully integrating these tools into inpatient environments. This is an acceptability and integration exploratory study conducted prior to the implementation of an ambient AI scribe in an ICU setting, in order to inform its future use by identifying key factors that may influence adoption. Our work contributes to human-computer interaction (HCI) research on AI in health care by grounding technology implementation and design in the lived experiences of those working on the front lines of care.

## Methods

### Ethical Considerations

This study was approved as exempt as a quality improvement initiative by the University of California, Los Angeles (UCLA) Office of the Human Research Protection Program (IRB-25‐0434). The study posed minimal risk, focused on system-level workflow improvements, and did not involve identifiable private information. All participants were informed of the interview purpose, participation was voluntary, verbal consent was obtained, and responses were deidentified for analysis. Appropriate measures were taken to protect participant privacy and confidentiality, and participants were not compensated. The study commenced in April 2025 and ended in July 2025. Semistructured interviews were guided by the RE-AIM/PRISM (Reach, Efficacy, Adoption, Implementation, and Maintenance/Practical, Robust Implementation, and Sustainability Model) framework. Thematic analysis was conducted using both inductive and deductive approaches.

### Setting, Study Population, and Recruitment

Given the distinct ways in which different clinicians may engage with an ambient AI scribe in the ICU, a diversity of perspectives was essential for our focus. All clinicians routinely involved in ICU patient care at all UCLA ICUs were eligible to participate, including physicians (attendings, residents, and fellows across disciplines), advanced practice practitioners (APPs), registered nurses, and respiratory therapists (RTs). We specifically sought to recruit providers other than doctors to broaden the perspective. The only exclusion criterion was affiliation with the Quality Improvement team. Individual demographic data (eg, age and sex) were not collected to protect the anonymity of participants in smaller subgroups, such as APPs. Clinicians were recruited via electronic mail messages and announcements at educational conferences. Participants were recruited across both Ronald Reagan UCLA Medical Center and Santa Monica UCLA Medical Center, 2 distinct hospitals within the UCLA Health System.

### Interviews and Focus Groups

The decision to interview clinicians individually or in a focus group was dictated by their availability to meet with study personnel. While focus groups were initially prioritized for larger clinician cohorts (eg, residents, ICU attendings, and ICU nurses), these were logistically difficult to conduct, necessitating interviews. For each interview, verbal consent was obtained prior to participation. Semistructured interviews, using an interview guide developed for this study (Multimedia Appendix 1), were conducted by a clinician trained in qualitative research methods, which enhanced contextual relevance and rapport with participants [13]. Reflexivity practices were employed throughout data collection to minimize bias and maintain analytic rigor. To ensure comparability across modes, we used the same semistructured guide in both settings and focused our analysis on participants’ experiences and perceptions rather than interactional features of group discussion.

UCLA Health had entered into an enterprise agreement with Nabla, which had been implemented in ambulatory clinics for over a year but had not yet been deployed in inpatient settings at the time of this study. At the start of the interview, the interviewer provided a live demonstration of the Nabla ambient AI scribe to illustrate how it could be used to assist with drafting a summary of a clinical discussion. This demonstration was intended to establish a consistent baseline of understanding across all participants, enabling them to offer informed perspectives on the potential use of ambient AI scribes for ICU documentation. Participants were asked a series of questions on common ICU structured discussions, using prompts aligned with the interview guide (Multimedia Appendix 1).

### Analysis Plan

Qualitative methods were used to explore trust and appropriate use of ambient AI scribes in the ICU [14,15]. Focus groups were facilitated by a trained clinician moderator, and audio recordings were transcribed and deidentified. Transcripts were not returned for member checking due to practical constraints. Sampling was guided by thematic saturation, with a target of 10 to 20 interviews per key clinician group involved in ICU communication. Given the small number of nurse practitioners and physician assistants, we sought to include the full cohort. Data collection continued until no substantively new themes emerged overall. Because some roles were underrepresented, saturation within individual groups cannot be assumed; findings, therefore, reflect cross-cutting rather than role-specific perspectives.

Transcripts were analyzed using a grounded theory approach, allowing themes to emerge from the data [16]. Two authors developed and iteratively refined a codebook (Multimedia Appendix 2), applying constant comparison and recoding transcripts as needed [16]. They independently coded transcripts and met regularly to reconcile differences and reach consensus. This consensus approach was chosen to prioritize depth of interpretation over interrater reliability metrics. Checklist 1 summarizes reporting elements for qualitative informatics studies included in this paper. Coding was facilitated using Atlas.ti (2025 ATLAS.ti Scientific Software Development GmbH).

## Results

### Clinician Perspectives (Frontline Providers)

From April 2025 to July 2025, we interviewed and conducted focus groups with 52 ICU clinicians (Table 1). Interviews ranged from 14 to 46 minutes in duration, with a median duration of 24 (IQR 17-34) minutes. Two focus groups were conducted with a total of 5 ICU trainees (residents and fellows), 1 focus group with a total of 2 ICU nurses, and 2 focus groups with a total of 4 RTs.

**Table 1.** Overview of study participants by role and data collection method.

Participant type	Data type, n	
	Interview	Focus group
Attending ICU^a^ physicians^b^	18	0
APPs^c^	5	0
ICU trainees (fellows/residents)	5	5
ICU nurses	7	2
ICU respiratory therapists	6	4
Total	41	11

a ICU: intensive care unit.

b ICU disciplines represented in interviews were surgical ICU, cardiothoracic ICU, cardiac ICU, medical ICU, neurocritical care ICU, liver ICU, pediatric ICU, and neonatal ICU.

c APP: advanced practice practitioner.

All clinician groups emphasized the importance of clear and accurate documentation during structured ICU discussions. There was broad agreement that capturing the content of team-based discussions was critical to safe and effective patient care, and that clinicians wanted these exchanges to be documented well and meaningfully:

*I personally care a lot about the progress notes and the accuracy and quality of the notes and so I spend quite a bit of time on them on a daily basis when I'm in ICU*.

[ICU attending 1]

*A discussion that I think would benefit from an ambient AI scribe is a goals-of-care conversation. These conversations can get very complex, very long, and sometimes it’s really hard to capture all the nuance. It would be helpful if that could be translated into a really high-quality note*.

[ICU attending 2]

*The thing with the handoffs is there’s no documentation whatsoever right now. There are a lot of details that you've discussed verbally, but probably should be in a note*.

[ICU trainee 1]

Even though structured team discussions were seen as critically important, clinicians identified several barriers and facilitators to documenting them effectively. Chief among these was time pressure, but other factors included patient care and teaching responsibilities, and the cognitive load associated with translating medically complex discussions into a note:

*Sometimes there’s just too much patient care- I frequently do notes at home … on a very busy day, as much as an hour or more at home*.

[ICU attending 3]

*There really is a group discussion … why we're thinking what we're thinking and why we're doing what we're doing … there’s definitely things lost in how that gets translated to the progress note*.

[ICU nurse 1]

*Those discussions are very rich. They’re very comprehensive … But the richness of the discussion frequently does not get translated into a note that I’m writing or sometimes even what the resident writes*.

[ICU attending 4]

Clinicians felt that the documentation burden detracted from patient care and trainee education responsibilities:

*I find it frustrating that writing notes takes so long. And that means there’s just less time to actually do bedside teaching, patient updates with family members, and such*.

[ICU attending 5]

*The most frustrating thing is that the documentation is important. However, it is not more important than actually being with the patients and doing clinical care. Every minute I spend writing my notes is time that I'm not spending at the bedside*.

[ICU attending 6]

From the interview themes, key supporting factors and barriers toward high-quality documentation during structured ICU discussions (Table 2) were identified. For each discussion type, participants described specific factors that either supported or hindered accurate information capture and documentation. We mapped interview responses to these facilitators and barriers to highlight the sociotechnical context in which ambient AI scribe technologies would operate.

We asked participants to share their thoughts on the role that ambient AI scribes might play in their workflows. Overall, clinicians expressed interest that ambient AI scribes could enhance consistency and completeness in documentation and enable more time for resident education and patient care, but did express reservations over issues of consent and concerns for surveillance (Table 3; Textbox 1).

**Table 2.** Supporting factors and barriers to effective documentation of structured team-based discussions, with illustrative quotes.

Type of structured discussion	Supporting factors	Barriers
Multidisciplinary ICU^a^ rounds	Structured rounding frameworks promote organized, systems-based documentation *“Our rounding discussion is very structured and systems-based; an [ambient AI^b^ scribe] could capture that and turn it into a clear assessment and plan.”* (ICU attending 7) EHR^c^ smartphrases and templates *“The auto-populate stuff is somewhat helpful, but it mainly pulls in objective data. Still, smartphrases and templates let us finish some notes in minutes.”* (ICU attending 3) *“You have to like good smartphrases if you want to document well.”* (ICU trainee 1) Professional expectation for daily documentation *“It’s kind of a painful process for me, but it also helps me to crystallize my thinking and be able to articulate my thoughts. The attending addendum helps me to synthesize in a very clear manner my assessment of the patient overall and the plan going forward.”* (ICU attending 4)	Time pressure *“The biggest one is time constraints … you try to do the bare minimum and leave, which then leaves gaps in documentation.”* (ICU attending 8) *“Most of the time I go home and I have not been able to complete my ICU notes for the day.”* (ICU attending 9) Note-writing interruptions disrupt cognitive flow during writing *“If a patient is crashing, there’s no way to get to documentation; most days I’m finishing at home, off the clock.”* (ICU attending 10) Competing clinical demands *“You either need to make a decision on whether you’re going to be taking care of a patient or focusing on your documentation.”* (ICU attending 9) *“I do need to carve out at least an hour, and that means that’s just less time to actually do bedside teaching. and patient updates with family members.”* (ICU attending 11) Information loss from the discussion *“But even as I’m writing the note and reading through it again, and trying to think- even after I’ve signed notes sometimes- I’ll remember something that I meant to write down or that we talked about and didn’t put in the note, and have to go back and change it again.”* (ICU attending 5)
Handoffs and transitions of care	OR^d^-to-ICU handoff checklist serves as a strong scaffold for documentation *“The CT^e^ and SICU^f^ OR-to-ICU handoff is highly structured; with a little training the scribe could create a note for it.”* (ICU attending 2)	Handoff documentation is not routinely practiced and may encounter resistance if perceived as adding to clinicians’ workload *“We don’t currently put handoffs in a note- is the handoff going to take more time if we have to verify a note?”* (ICU attending 4)
Goals-of-care discussions	Documenting changes in a patient’s code status is an expected standard of care *“The goals-of-care conversation would be a prime target for the [ambient AI scribe].”* (ICU trainee 2)	Nuanced clinical conversations are often difficult to fully capture in written documentation *“These conversations are really complex. Our ability to write out the nuances of these conversations efficiently and effectively isn’t always possible.”* (ICU attending 13)

a ICU: intensive care unit.

b AI: artificial intelligence.

c EHR: electronic health record.

d OR: operating room.

e CT: computed tomography.

f SICU: surgical ICU.

**Table 3.** Clinician-identified human factors for ambient artificial intelligence scribe implementation in intensive care unit settings.

Theme	Key insight	Representative quote
Lack of education and training	Clinicians feel unprepared to work with AI^a^ technologies	*“AI is coming for all of our futures … we’re going to be using AI technology, but no one is teaching us anything about it.”* (ICU^b^ trainee 3) *“The health literacy or the literacy around AI is so poor right now… there’s misinformation and not much familiarity.”* (ICU trainee 2)
Need for consent and transparency	Clinicians emphasize the importance of informing the team before using ambient AI scribes	*"It should be like a standard practice- you would say at the beginning: ‘We’re going to be using the [ambient AI scribe]. Is everyone OK with that?’"* (ICU nurse 3) *“Whoever is using it should notify everybody that it’s on and how it’s going to be used.”* (ICU nurse 4) *“There’s consent fatigue … but there’s still a need to present it elegantly and transparently.”* (ICU trainee 4)
Concern for surveillance and psychological safety	Clinicians voiced concerns about surveillance stemming from a lack of clarity regarding how ambient AI scribe data would be used	*“What’s actually being kept? Is it an audio recording, a transcript? Is it a summary? I don’t understand what is being kept from the scribe.”* (ICU RT^c^ 1) *“You’re being watched… and that changes your behavior. It’s like the panopticon.”* (ICU trainee 1) *“Providers should be protected from surveillance.”* (ICU trainee 5)
Communication adaptation in response to ambient AI scribe	Staff may shift language to accommodate the ambient AI scribe	*“I think we’re going to adapt how we speak during rounds so that the [ambient AI scribe] can do its best job.”* (ICU attending 12) *“It will increase professional descriptions and decrease off-the-cuff ridiculous remarks. People actually need to use more formal medical language. Instead of ‘AKI^d^,’ the patient has ‘prerenal acute kidney injury.’”* (ICU attending 1) *“People would be more cautious. they might consider their word choice more carefully.”* (ICU nurse 5) *“We might structure our communication a little more differently … if we want to make it a very structured note, I bet we would adhere to that even more.”* (ICU attending 15) *“It could affect the frankness of how someone describes a case.”* (ICU RT 2)
Perceived reduction in cognitive load	Ambient AI scribe may free cognitive resources during rounds	*“It’s going to help me just pay attention to the conversation without having to frantically jot down notes at the same time.”* (ICU APP^e^ 1)
Preference for personalized output and the need for review	Clinicians want editable summaries that are tailored to specific discussions	*"The most important would be accurate transcription … the second most important feature would be that it then organizes that information into some type of paragraph or systems-based note template that I could then review and edit.”* (ICU attending 7) *“There’s no way I would want it to automatically put in a note without me reviewing it.”* (ICU attending 16)
Opportunity to capture structured communication exchanges that are inconsistently or not documented at all	Ambient AI scribes could document key conversations often missed in the chart	“*I do think using it for the goals-of-care conversation would be nice. Sometimes the notes on these discussions are sparse and you don’t know why patients went from Full Code to DNR*^f^.” (ICU nurse 6) “*I don’t think we really know how people are using the OR*^g^*-to-ICU handoff checklist. If you have to place a note now, people will have to use it*.” (ICU trainee 2)

a AI: artificial intelligence.

b ICU: intensive care unit.

c RT: respiratory therapist.

d AKI: acute kidney injury.

e APP: advanced practice practitioner.

f DNR: do not resuscitate.

g OR: operating room.

Textbox 1. **Clinician perspectives on implementing ambient artificial intelligence scribes for documenting structured communication exchanges in the intensive care unit.**

**In support of ambient artificial intelligence (AI) scribe implementation**

“*I think notes are going to be more accurate and more timely or I should say more thorough documentation of the things that we talk about on rounds*.” (ICU attending 4)

“*Having an [ambient AI scribe] that's reviewed by a doctor might actually lead to more accurate documentation*.” (ICU attending 5)

“*We would also be able to produce an assessment and plan a bit more systematically than we've been doing … for the dual purpose of communicating and educating our trainees and nurses and the [ambient AI scribe]*.” (ICU attending 6)

“*Hopefully, if this is done right, you're spending more time with patients, you're spending more time doing care and teaching, you're spending less time billing, coding, and writing notes*.” (ICU attending 7)

“*The OR-to-ICU handoff has no documentation currently. This could help*.” (ICU attending 9)

“*It’s going to help the doctors. Sometimes their notes have too much copy and paste*.” (ICU nurse 1)

"*You have to build your relationship. The AI has to learn you and you have to learn the AI*.” (ICU attending 16)

**Skeptical of ambient AI scribe implementation**

“*If it can provide a clear summary with minimal editing- yes I would use it. If not, I probably wouldn’t use it*.” (ICU attending 17)

“*I would have to edit it … I never trust an AI. How accurate is it?*” (ICU attending 16)

“*You’re being watched … when you are being watched, your behavior absolutely changes*.” (ICU trainee 6)

“*No one trusts anything anymore… you're still recording verbatim what is being said, even if you generate a summary*.” (ICU trainee 1)

“*Not everybody is comfortable being recorded … some people might think it's surveillance*.” (ICU nurse 8)

“*I do hope that it is able to recognize people with different accents and different background language. My concern would be that I have an accent and it would pick up my words in a different way*.” (ICU attending 18)

“*Sometimes family members are present on rounds … I don't know how they would feel about an ambient AI scribe*.” (ICU nurse 4)

“*There's a lot of noise. So I, you know, I could see things being potentially incorrectly transcribed because there's like a lot just, it's just like so much going on*.” (ICU attending 15)

*I think my dream would be that … I use this scribe during rounds. When I am done rounding and seeing my patients, I go back to the computer and I already have a templated progress note draft for me that I make some minor edits to before signing into the medical record*.

[ICU attending 1]

*I think recording always affects the freedom of speech … If you feel like you're being watched when you're discussing a patient’s care … not everything may come out that freely*.

[ICU trainee 1]

### Stakeholder Perspectives From ICU Team Members

While our interview guide was developed with the assumption that ambient AI scribes would support ICU attending physician documentation, we also sought to capture perspectives from other ICU team members. These included individuals who regularly participate in structured communication discussions in the ICU, all of whom would be exposed to the presence of an ambient AI scribe. We conducted role-specific thematic analysis to examine perspectives by clinical role. Representative themes are summarized below:

- ICU trainees (residents and fellows): trainees expressed openness to piloting the tool in ICU workflows. They perceived that AI tools would become increasingly central to their practice and expressed a clear need for training toward AI literacy and integration into clinical education. They expressed concerns over data governance and the potential for trainee surveillance.
- ICU APPs: APPs describe frequent gaps between what is discussed during ICU rounds and what is captured in progress notes, often due to time constraints and rounding fatigue. They viewed ambient AI scribes as a means to allow them to focus on listening instead of documenting during real-time discussions. However, they expressed concerns about overreliance and transcription errors, particularly with soft-spoken or accented speakers, which might require extra correction.
- ICU registered nurses: nurses were generally supportive of the use of ambient AI scribes in physician documentation workflows, particularly if transparency and consent protocols were established. Some also expressed interest in adapting the tool for nursing documentation, such as end-of-shift summaries, provided they had the ability to review and edit the outputs.
- ICU RTs: RTs supported technologies that improve documentation and communication quality. They did not anticipate major workflow disruptions from ambient AI scribes and felt that improved documentation might indirectly enhance interdisciplinary comprehension.

## Discussion

### Principal Findings

We synthesize these findings to highlight implications for the implementation and policy of ambient AI scribes in inpatient settings. ICU clinicians identified common structured communication discussions, such as daily multidisciplinary rounds, handoffs and transitions of care, and goals-of-care discussions, as high-value opportunities where ambient AI scribes could meaningfully improve documentation. Documentation of these discussions is often compromised in quality or goes undocumented entirely as a result of time constraints, cognitive load, and the demands of direct patient care. Clinicians perceived that this technology might reduce the burden of documentation, allowing teams to focus on the conversation and develop a clearer shared mental model of the patient. This aligns with longstanding insights from HCI, which emphasize the importance of reducing cognitive load and supporting task-focused attention when implementing new technology in high-stakes clinical environments [17].

Ambient AI scribes were viewed not only as tools to reduce documentation burden but also as infrastructure for reinforcing and sustaining real-world standardized communication practices in the ICU [18]. Clinicians perceived that ambient AI scribes might enhance patient safety by documenting critical information that might otherwise be lost, as was notably discussed in the case of handoffs for which no formal documentation exists. Team members believed that capturing undocumented discussions could enable Quality Improvement leaders to measure and monitor real-world handoff practices. This reflects a broader theme in the HCI literature: technology not only can support workflows but also sustain standardized practices over time [19,20]. However, there was recognition that ambient AI scribes would only have an impact in this regard as long as there was institutional commitment to defining and requiring standardized communication and documentation practices that ambient AI scribes can reliably support. Without this foundational structure of expected communication frameworks with their associated required documentation, clinicians felt that ambient AI scribes might not be used for specific discussions that could benefit from documentation, such as handoffs.

A key theme across interviews was clinicians’ strong sense of responsibility for the accuracy of ambient AI scribe output, aligning with prior research on trust in automation [21]. Participants emphasized the need to review and edit draft notes before attestation and viewed themselves as the final arbiters of documentation quality. This sense of accountability was a key factor in their willingness to adopt the technology. Few participants expressed concern that clinicians would neglect the task of editing and instead assumed that they would invest the necessary effort to ensure accuracy. They noted that if the draft output required excessive editing, it could add additional work that would undermine the technology’s value proposition of saving time. They anticipated that each clinician would develop strategies to balance efficiency and accuracy. These insights suggest that successful adoption depends not only on the system’s baseline accuracy but also on clinicians’ ability to learn how to work effectively with the technology.

Another theme was the need to tailor ambient AI scribe output to diverse clinical documentation needs, consistent with the HCI concept of technology appropriation and “corealization” [22-24]. Participants felt that output formats must be co-designed with end users and adapted to the clinical context. For example, preferences for documenting daily ICU rounds varied by attending: some favored concise narrative summaries, while others preferred structured formats aligned with service-level templates. Goals-of-care conversations were viewed as best captured in a narrative paragraph that conveyed the rationale behind code status decisions. The OR-to-ICU handoff, which is a highly structured discussion that adheres to site-specific handoff checklists, was desired to be documented in a structured form similar to its associated checklist. Finally, some clinicians wished to co-design the output iteratively as they gained experience with the tool. These findings underscore the importance of participatory design approaches that give clinicians agency in developing how ambient AI-generated documentation is presented [25]. One potential limitation is whether ambient AI scribe vendors can support this functionality; however, as these tools become more prevalent in inpatient settings, close collaboration with vendors will be essential to ensure outputs are appropriately tailored to clinical context. This aligns with core principles of human factors engineering, which emphasize that successful health IT must fit seamlessly within existing work patterns at the individual, team, and institutional levels [26].

The interest in trialing ambient AI scribes in the ICU was tempered by concerns about the sociotechnical implications of voice-based AI in team-based, high-risk environments, highlighting the need for careful, context-sensitive implementation [27]. Many participants felt that the presence of an ambient AI scribe could alter ICU team communication, although mostly in a positive way. Some clinicians anticipated a need to “narrate” more explicitly, for instance, by speaking directly to the ambient AI scribe, but perceived that this might encourage clearer, more intentional communication, potentially enhancing both the educational value of rounds and the quality of documentation. These themes mirror broader debates in HCI and sociotechnical systems research about how data capture can influence professional behavior [28-30]. Concerns were also raised regarding the transparency of data use and the challenges of obtaining consent within dynamic ICU communication workflows. Given the team-based nature of ICU rounds and other collaborative discussions, clinicians emphasized the importance of ensuring that all participants are aware when an ambient AI scribe is in use. Participants acknowledged that rounding discussions are often interrupted by consulting team members entering and exiting the conversation to provide recommendations or are interrupted by family members who may enter mid-discussion. Some expressed concern about the feasibility of having the ambient AI scribe’s end user consistently deliver verbal consent language in such dynamic environments, highlighting the potential need for hardware-based solutions, such as visual indicators, to clearly signal when recording is active. Nevertheless, incorporating standardized consent language into structured discussion workflows, such as verbal announcements at the start of a discussion, was felt to be vital in clarifying the functions of the ambient AI scribe, how its outputs will be used, and what data will be retained. Although participants acknowledged the potential of ambient AI scribes to improve documentation, their use in provider-provider discussions raised concerns about constant job performance surveillance. Participants emphasized that education on data governance will be critical to building trust and positioning these tools as supportive rather than evaluative.

Based on themes garnered from the interviews, a list of implementation considerations for ambient AI scribes in inpatient workflows was generated, as shown in Textbox 2. These considerations were directly informed by the insights and concerns raised by ICU clinicians and represent needs across different roles and use cases. These recommendations aim to serve as a pragmatic guide for health systems that wish to implement pilot testing of ambient AI scribes in inpatient workflows.

Textbox 2. **Implementation considerations for integrating ambient artificial intelligence scribes into in-patient clinical workflows.**

**Context-aware implementation**: Identify team-based discussions for which the use of an ambient artificial intelligence (AI) scribe would address a clear documentation need, or where documentation could improve quality and safety.

**Customization of scribe output**: Co-design with ambient AI scribe vendors to ensure the output conforms with desired formats.

**Medico-legal responsibility**: Develop institutional guidance on editing and verification workflows, and educate clinicians on their medico-legal responsibility for the accuracy of ambient AI-generated documentation.

**Trust, transparency, and data governance**: Clearly communicate that ambient AI scribes are intended to support documentation efficiency, with transparent policies on when recording occurs, what is stored, and how data are protected.

**Workforce outreach**: Demonstration of ambient AI scribe functionality and discussion with team members may foster understanding and comfort.

**Multiprovider consent**: Establish clear protocols for obtaining consent from all participating providers before initiating ambient AI scribe during team-based discussions.

**Training and support**: Provide training and support to ensure effective use and foster understanding of AI scribe functionality.

**Impact measurement**: Define metrics to assess effects on documentation quality, workflow, and safety, supported by ongoing feedback and refinement.

Our study has notable strengths. A key strength of this study is the inclusion of perspectives from a range of ICU stakeholders who would directly engage with an ambient AI scribe. The use of qualitative methodology of a diverse set of ICU team members captured a more holistic view of how ambient AI scribes may impact team dynamics and might alleviate documentation challenges of important team-based discussions, offering insights to guide future implementation and evaluation. Second, several data sources (interviews, focus groups) to support the findings were used.

### Limitations

The study has important limitations. First, because ambient AI scribes were not yet in routine clinical use in the inpatient setting, participants’ views were based on anticipated rather than observed use. This was intentional, to proactively inform how it would be used in practice, in line with recommendations to embed ethical study into implementation processes [31]. A second limitation of this study is the absence of patient and family perspectives. While this study focused on ICU staff and clinicians to address its specific research aims, patients and families may hold distinct concerns and perspectives that are not captured here and represent an important area for future research. Finally, the live demonstration of the Nabla ambient AI scribe highlighted key functionalities (automatic speech recognition and output formatting options), but participants’ responses may have been influenced by priming effects associated with exposure to the demonstration.

### Future Directions

Future research should evaluate ambient AI scribe tools in real-world ICU settings using prospective, pragmatic study designs. Such studies should examine their impact on interprofessional communication quality, how clinical teams communicate in the presence of an ambient AI scribe, documentation completeness, and clinician well-being across professional roles. Relevant metrics may include communication fidelity scoring (eg, the presence of structured discussion elements), professional fulfillment or burnout indices, and provider-level indicators such as EHR audit-log data and time spent in notes. Future work could also focus on the perspectives of family members and patients exposed to an ambient AI scribe during an ICU hospitalization.

Understanding clinicians’ perceptions of how new technologies could be used to improve the quality of care delivery, as reported here, is emblematic of the type of investigation needed to make meaningful advances in health care value. The work of sociologists and patient safety experts, such as Dixon-Woods and Bosk, has emphasized the importance of understanding health care culture and the interactions among diverse professionals who must communicate effectively to achieve optimal outcomes [32]. Nowhere is this more evident than in the ICU, where structured team-based discussions are essential but suffer from documentation challenges. Ambient AI scribes may potentially improve how these discussions are captured, but understanding this requires a multidisciplinary approach. As with prior works that have examined structured discussions through collaborative lenses [33], our study aims to extend this tradition by exploring how ambient documentation tools might align with existing team-based communication practices to enhance safety and quality in ICU care delivery.

### Conclusion

Ambient AI scribes have emerged as promising tools to alleviate documentation burden, but their integration into high-stakes settings like the ICU demands a deeply human-centered approach. Through interviews with ICU clinicians, we uncovered optimism about the potential of these tools to assist with the documentation of structured team-based communication workflows, but clinicians emphasized the need for transparency in data governance. Our findings highlight the sociotechnical complexities of deploying ambient AI in safety-critical inpatient environments and suggest that success hinges not only on technological performance but also on thoughtful integration into clinical practice, addressing clinician concerns about transparency, trust, and the potential for perceived surveillance. We offer implementation suggestions that prioritize transparency and clinician control, contributing to broader HCI conversations on AI-assisted documentation and human-AI collaboration in health care.

## Supplementary material

10.2196/81445 Multimedia Appendix 1 Interview script for frontline clinicians (intensive care unit [ICU] attendings, ICU fellows/residents, ICU nurses, ICU respiratory therapists, ICU advanced practice providers).

10.2196/81445 Multimedia Appendix 2 Final codebook with definitions.

10.2196/81445 Checklist 1 COREQ checklist.

## Abbreviations:

AI: artificial intelligence
APP: advanced practice practitioner
COREQ: Consolidated Criteria for Reporting Qualitative Research
EHR: electronic health record
HCI: human-computer interaction
ICU: intensive care unit
RE-AIM/PRISM: Reach, Efficacy, Adoption, Implementation, and Maintenance/Practical, Robust Implementation, and Sustainability Model
RT: respiratory therapist
UCLA: University of California, Los Angeles
