# Supplementary material for: Clinician Perspectives on Ambient AI Scribes in the Intensive Care Unit: Qualitative Interview Study
Source: JMIR Med Inform. 2026 Jul 2;14:e81445. doi: 10.2196/81445 (PMC13325621; doi:10.2196/81445)
Supplement: Multimedia Appendix 1 [file medinform-v14-e81445-s001.docx]

| **Multimedia Appendix 1. Interview Script for Frontline Clinicians (ICU Attendings, ICU fellows/residents, ICU Nurses, ICU Respiratory Therapists, ICU Advanced Practice Providers).** |
| --- |
| **Introductory Language:**  We are exploring how ICU attending physicians might use ambient AI scribes, tools that passively listen to team conversations and generate real-time text summaries, to support documentation of structured communication activities such as ICU rounds, handoffs and transitions of care, and goals-of-care discussions. We want to understand the current pain points, workarounds, and inefficiencies in ICU documentation workflows, and to explore how emerging technologies like ambient AI scribes might help address these challenges or reshape existing practices. We are particularly interested in how such tools could influence interprofessional communication, documentation quality, and care delivery in the ICU. This interview is being audio recorded so that we can create a written transcript later for data analysis. When we transcribe the interview, we will not use your name, and nothing you say will be linked to your identity. You can decline to answer any question at any time.  Before we begin, I’ll show you a demonstration of how an ambient AI scribe captures and summarizes clinical conversations in real time [*demonstrate ambient AI scribe use prior to start of interview*].  Is it okay to proceed? |
| Question/Prompt   - Probe |
| What kind of clinician are you?  How long have you been working in your current capacity?  1. Perceived Need and Pain Points   - Please tell us about a time when ICU documentation felt especially frustrating.   - What made that situation difficult?   - How did it affect your ability to care for patients or collaborate with the team?   - Were there specific tools or systems that contributed to that frustration? - What makes it hard to document effectively during a busy shift?   - Are there specific times of day, types of patients, or types of documentation (e.g., notes, handoffs, checklists) that are particularly challenging?   - How do competing responsibilities (e.g., direct patient care, teaching, managing the team) impact your documentation workflow? - How do gaps in physician documentation affect your ability to do your job?   - Can you give an example where incomplete or missing documentation created confusion or led to communication breakdowns?   - What workarounds do you typically use when documentation is delayed or insufficient?   - How do these gaps affect patient safety or team coordination?   2. Familiarity and Attitudes Toward Ambient AI Scribes   - What do you imagine could be helpful about using an ambient AI scribe that listens during rounds? - What worries or concerns might you have about using a voice-based AI scribe in the ICU?   3. Workflow Fit, Team Dynamics, and Equity   - How do you think an ambient AI scribe might change how ICU rounds happen? - Do you think some team members might benefit more or be negatively impacted by this tool? - Could having an ambient AI scribe make people more or less comfortable speaking up during rounds?   4. Other Structured Communication Exchanges   - What types of ICU documentation do you think an ambient AI scribe might be especially helpful for? - Are there other conversations where you think this kind of tool could help?   5. Trust, Safety, and Autonomy   - How important is it to you to be able to review or edit the AI-generated documentation before it’s saved? - Can you imagine a scenario where you wouldn’t want an ambient AI scribe to be used?   6. Evaluation and Success Metrics   - What would convince you that this tool is worth using regularly? - How quickly would you expect to notice if this tool is helping or causing problems?   7. Consent and Ethical Considerations   - Should team members be asked for consent before an ambient AI scribe is used? - How should patients and families be told that an AI tool may be listening in on parts of their care?   8. Closing / Reflective   - Is there anything else you want the Quality Improvement team to understand before moving forward with this technology? |
