# Supplementary material for: Clinician Perspectives on Ambient AI Scribes in the Intensive Care Unit: Qualitative Interview Study
Source: JMIR Med Inform. 2026 Jul 2;14:e81445. doi: 10.2196/81445 (PMC13325621; doi:10.2196/81445)
Supplement: Multimedia Appendix 2 [file medinform-v14-e81445-s002.docx]

**Multimedia Appendix 2. Final Codebook with Definitions**

| **Code Name** | **Definition** |
| --- | --- |
| CurrentDocumentationQuality | Any mention of what current documentation looks like as a whole. Does not include any mention of the content that should be documented. |
| DocumentationImportance | Any mention about whether or not participants consider documentation worthwhile. Includes discussion of peer views as well. |
| TimeConstraints | Any mention of not having enough time, nonclinical time, dedicated time, including coordinating a time, working around rotation schedules. |
| NoteWritingTimeBurden | Any mention of perceived length of time required to complete ICU documentation during the shift. |
| AfterHoursWork | Any mention of completing documentation tasks at home or off-the-clock. |
| SystemsBasedTemplateStrain | Any mention of difficulty completing mandated systems-based assessment and plan formats. |
| CopyForwardErrors | Any mention of inaccurate or outdated content propagated via copy-paste. |
| RoundsToNoteInformationLoss | Any mention of nuances from rounding discussion not captured in the written note. |
| BillingDrivenDocumentation | Any mention of pressure to include exhaustive systems-based language for coding and billing. |
| MultiSourceIntegrationNeed | Any mention of the challenge of incorporating data from multiple sources (nurses, family, labs) into a single coherent note. |
| TimeVsPatientCareTension | Any mention of documentation time detracting from time at the patient bedside. |
| CompetingClinicalDemands | Any mention of how note-writing is disrupted by urgent clinical tasks or new admissions. |
| EHRSmartPhraseSupport | Any mention of the use of Epic templates and smartphrases as current facilitators to documentation. |
| StructuredRoundingFrameworks | Any mention of the use of systems-based verbal rounding structures that may be helpful for ambient AI capture. |
| NeedToReviewAIOutput | Any mention regarding an expectation that clinicians must review and verify all AI-generated documentation. |
| PrivacyConsentConcern | Any mention about concerns about surveillance, recording, or need for multi-speaker consent. |
| SpeechBehaviorChange | Any mention of any general change in communication behavior (such as more cautious language) when ambient AI scribe is active. |
| AccentAndSpeakerIDAccuracy | Any mention of concerns that AI will misidentify speakers or mis-transcribe accents. |
| BackgroundNoiseInterference | Any mention of ICU environmental noise (e.g., alarms, airflow) that may interfere with transcription accuracy. |
| ProfessionalLanguageUplift | Any mention of the presence of ambient AI scribes encouraging clearer or more formal diagnostic language. |
| TeachingEnhancement | Any mention of AI scribe use promoting better verbalization of reasoning and treatment rationale, benefiting learners. |
| TeachingDeferral | Any mention of attendings delaying teaching to avoid recording informal comments. |
| CompetingTeachingDemands | Any mention of how note-writing is disrupted by resident teaching responsibilities. |
| GoalsOfCareSuitability | Any mention of AI scribe being seen as valuable for summarizing complex family or GOC discussions. |
| ORToICUHandoffSuitability | Any mention of AI scribe being used for structured surgical OR-to-ICU handoffs. |
| CognitiveLoadRelief | Any mention of AI scribe reducing documentation burden and allowing clinicians to focus on listening. |
| QualityImprovementPotential | Any mention of AI scribe transcripts being used for auditing communication or quality of care. |
| SignatureLiability | Any mention of clinicians expressing concern that signing AI-generated notes implies legal accountability. |
| DesireForMeasurableTimeSavings | Any mention of desire for clear, quantifiable time-saving or efficiency metrics to justify ambient AI scribe adoption. |
| EquityAndTeamDynamics | Any mention of concerns about how ambient AI scribe use might affect who feels comfortable speaking and overall communication dynamics. |
| InaccuracyAndOverreliance | Any mention of concerns about blind trust in AI-generated notes and reduced vigilance in checking the scribe output. |
| NoteWritingExpectationAsScaffolding | Any mention of how the expectation to write a daily note facilitates reflection, thoroughness, synthesis, or structured thinking. |
| CommunicationStandardization | Any mention of how the presence of the AI scribe might result in more standardized communication. |
| MentalModelClarity | Any mention of ambient AI scribes improving team members’ shared understanding or mental model of the patient. |
| NarrationPrompting | Any mention of clinicians adjusting their speech (e.g., “narrating” more explicitly) to guide or enhance AI-generated documentation. |
| EditingBurdenVsTimeSavings | Any mention of clinicians weighing the time saved against the time spent reviewing and editing draft outputs. |
| VisualConsentIndicators | Any mention of hardware or interface features (e.g., lights) to visibly signal when recording is active to support transparency and psychological safety. |
| RoutineConsentIntegration | Any mention of integrating standard verbal consent language into clinical workflows to inform participants about ambient AI scribe use. |
| PsychologicalSafetyImpact | Any mention of how ambient AI scribe use might affect team members' comfort, willingness to speak, or perceived surveillance. |
| ParticipatoryDesignImperative | Any mention of the need to involve end-users in designing or refining the technology to ensure clinical relevance and usability. |
| OrganizationalContextSensitivity | Any mention of needing ambient AI scribe systems to adapt to the unique workflows, cultures, or policies of specific inpatient settings. |
| ReadinessForAdoption | Any mention of site- or clinician-level readiness factors that would influence the success of implementation (e.g., training, prior exposure to similar tools). |
| OutputControlAndCustomization | Any mention of the desire for clinicians to personalize, co-design, or iteratively refine scribe-generated outputs based on evolving needs. |
| InstitutionalStandardizationNeed | Any mention of the need for institutional definitions of communication frameworks (e.g., rounding structure, handoff protocols) that ambient AI scribes should align with. |
| AmbientAsWorkflowInfrastructure | Any mention of ambient AI scribes serving not just as documentation tools but as a mechanism to reinforce or institutionalize structured communication practices. |
| TrustInAutomation | Any mention of trust or mistrust in AI-generated documentation, including expectations of output accuracy or skepticism. |
