# Supplementary material for: Clinician Perspectives on Ambient AI Scribes in the Intensive Care Unit: Qualitative Interview Study
Source: JMIR Med Inform. 2026 Jul 2;14:e81445. doi: 10.2196/81445 (PMC13325621; doi:10.2196/81445)
Supplement: Checklist 1 [file medinform-v14-e81445-s003.docx]

**Multimedia Appendix 3. COREQ Checklist.**

| **Checklist Item** | **Guide Questions** | **Fulfilled or Addressed?** | **Evidence/Comments** |
| --- | --- | --- | --- |
| 1. Interviewer/facilitator | Which author/s conducted the interview or focus group? | Yes | Stated in Methods, Interviews and Focus Groups section |
| 2. Credentials | What was the researcher’s credentials? | Yes | Stated in Methods, Interviews and Focus Groups section; also seen in author list on title page |
| 3. Occupation | What was their occupation at the time of the study? | Yes | Stated in Methods, Interviews and Focus Groups section |
| 4. Gender | Was the researcher male or female? | Yes | Interviews were conducted by one female and one male clinician researcher trained in qualitative methods. |
| 5. Experience and training | What experience or training did the researcher have? | Yes | Stated in Methods, Interviews and Focus Groups section |
| 6. Relationship established | Was a relationship established prior to study commencement? | Yes | Stated in Methods, Interviews and Focus Groups section |
| 7. Participant knowledge of the interviewer | What did the participants know about the researcher? | Yes | Stated in Methods, Interviews and Focus Groups section; obtained verbal informed consent, which stated research purpose |
| 8. Interviewer characteristics | What characteristics were reported about the interviewer/facilitator? | Yes | Stated in Methods, Interviews and Focus Groups section |
| 9. Methodological orientation and Theory | What methodological orientation was stated to underpin the study? | Yes | Stated in Methods, Analysis Plan section; grounded theory approach |
| 10. Sampling | How were participants selected? | Yes | Stated in Methods, Interviews and Focus Groups section; based on provider availability and self-identification, so convenience |
| 11. Method of approach | How were participants approached? | Yes | Stated in Methods, Study Population and Recruitment section; e-mails and announcements |
| 12. Sample size | How many participants were in the study? | Yes | Stated in Results, Clinician Perspectives section section |
| 13. Non-participation | How many people refused to participate or dropped out? Reasons? | No | Participation was self-selected; refusal rates were not tracked, non-participation cannot be quantified, and no participants withdrew. |
| 14. Setting of data collection | Where was the data collected? | Yes | Stated in Methods, Interviews and Focus Groups section |
| 15. Presence of non-participants | Was anyone else present besides the participants and researchers? | Yes | Stated in Methods, Interviews and Focus Groups section; nobody else present |
| 16. Description of sample | What are the important characteristics of the sample? | No | Stated in Methods, Study Population and Recruitment section; individual clinician demographic data are not available |
| 17. Interview guide | Were questions, prompts, guides provided by the authors? Was it pilot tested? | Yes | Interview script provided as Supplementary Table 1 |
| 18. Repeat interviews | Were repeat interviews carried out? If yes, how many? | No | Stated in Methods, Interviews and Focus Groups section; subjects each participated in one interview or focus group |
| 19. Audio/visual recording | Did the research use audio or visual recording to collect the data? | Yes | Stated in Methods, Interviews and Focus Groups section |
| 20. Field notes | Were field notes made during and/or after the interview or focus group? | Yes | Stated in Methods section; field notes taken during interviews/focus groups |
| 21. Duration | What was the duration of the interviews or focus group? | Yes | Stated in Results, Clinician Perspectives section |
| 22. Data saturation | Was data saturation discussed? | Yes | Stated in Results, Clinician Perspectives section; reached by consensus of researchers |
| 23. Transcripts returned | Were transcripts returned to participants for comment and/or correction? | No | Stated in Methods, Interviews and Focus Groups section |
| 24. Number of data coders | How many data coders coded the data? | Yes | Stated in Methods, Analysis Plan section; two authors coded |
| 25. Description of the coding tree | Did authors provide a description of the coding tree? | Yes | Codebook provided as Supplementary Table 1 |
| 26. Derivation of themes | Were themes identified in advance or derived from the data? | Yes | Stated in Methods, Analysis Plan section; authors developed codebook by reading through data, themes subsequently derived |
| 27. Software | What software, if applicable, was used to manage the data? | Yes | Stated in Methods, Analysis Plan section |
| 28. Participation checking | Did participants provide feedback on the findings? | No | Stated in Methods, Interviews and Focus Groups section |
| 29. Quotations presented | Were participant quotations presented to illustrate the themes/findings? Was each quotation identified? | Yes | Quotations, along with clinician subject indicators, provided in Results, Clinician Perspectives section |
| 30. Data and findings consistent | Was there consistency between the data presented and the findings? | Yes | Data presented and described in Results are consistent with findings relayed in Results and Discussion |
| 31. Clarity of major themes | Were major themes clearly presented in the findings? | Yes | Discussed in Results, Clinician Perspectives section and further described in Table 2 |
| 32. Clarity of minor themes | Is there a description of diverse cases or discussion of minor themes? | Yes | Discussed in Results, Clinician Perspectives section and Contextual Data section, Table 3, and Table 4 |

30/32 Addressed; Remaining 2 deemed not relevant to this study
